# Adipocyte FGF21 Signaling Defect Aggravated Adipose Tissue Inflammation in Gestational Diabetes Mellitus

**DOI:** 10.3390/nu16223826

**Published:** 2024-11-07

**Authors:** Lun Hua, Yi Yang, Haoqi Zhang, Xuemei Jiang, Chao Jin, Bin Feng, Lianqiang Che, Shengyu Xu, Yan Lin, De Wu, Yong Zhuo

**Affiliations:** 1 Animal Nutrition Institute, Sichuan Agricultural University, Chengdu 611130, China; hualun@sicau.edu.cn (L.H.); 71310@sicau.edu.cn (X.J.); linyan@sicau.edu.cn (Y.L.);; 2 Key Laboratory for Animal Disease-Resistant Nutrition of the Ministry of Education of China, Sichuan Agricultural University, Chengdu 611130, China; 3 Key Laboratory of Animal Disease-Resistant Nutrition of Sichuan Province, Sichuan Agricultural University, Chengdu 611130, China

**Keywords:** gestational diabetes mellitus, adipose tissues inflammation, FGF21, Tregs, PGE2

## Abstract

Gestational diabetes mellitus (GDM) is associated with increased inflammation in adipose tissues. Fibroblast growth factor 21 (FGF21) is an endocrine hormone which signals to multiple tissues to regulate metabolism. However, its role in GDM remains largely unknown. In this study, we found that impaired FGF21 signaling in GDM correlates with worsened inflammation and insulin resistance in white adipose tissues in mice. Mechanistically, the pregnancy-related upregulation of FGF21 signaling in adipocytes promotes the differentiation of regulatory T cells (Tregs), which are critical for reducing pregnancy-induced adipose tissue inflammation. The anti-inflammatory effects of FGF21 may involve linolenic acid-mediated PGE2 synthesis in adipocytes. These findings underscore FGF21’s role in mediating crosstalk between mature adipocytes and immune cells in white adipose tissue and suggest that targeting FGF21 signaling and its downstream metabolites could offer a potential therapeutic approach for GDM in humans.

## 1. Introduction

The global obesity epidemic among fertile women, coupled with rising rates of gestational diabetes mellitus (GDM), imposes a sizeable financial burden upon modern healthcare systems [1]. GDM increases the risk of preterm delivery, neonatal hypoglycemia, hyperbilirubinemia, and future cardiometabolic diseases for both mothers and offspring [2,3]. Pregnancies affected by GDM are characterized by chronic inflammation that promotes insulin resistance and induces endothelial dysfunction, increasing the risk of developing conditions such as type 2 diabetes, obesity, and cardiovascular diseases [2,3]. Adipose tissue (AT) is not only an energy reservoir, but it is also critical for systemic homeostasis via secretion of hormone-like adipokines. Thus, dysfunctional adipocytes are a major factor in the development of insulin resistance and type 2 diabetes (T2D) [4]. During pregnancy, adipose tissue adapts to expand and provide nutrients for embryonic growth and lactation, but this process is disrupted in obese and GDM pregnancies [5,6], with the underlying mechanisms largely unexplored.

During healthy pregnancies, gestational fat storage occurs through adipocyte hypertrophy [5]. However, obese women already have hypertrophied adipocytes prior to pregnancy, leading to greater insulin resistance than their lean counterparts [6]. This resistance diminishes insulin’s ability to suppress adipocyte lipolysis, resulting in fatty acid spillover, which exacerbates insulin resistance and promotes macrophage accumulation and inflammation in adipose tissue [6]. The increased presence of immune cells and pro-inflammatory molecules further worsens insulin resistance [7,8]. Developing pharmacological strategies to counter GDM requires a comprehensive understanding of these mechanisms, particularly the adaptive changes in maternal metabolism.

Fibroblast growth factor 21 (FGF21) is synthesized in multiple tissues, including the liver, pancreas, and adipose tissue. However, under normal physiological conditions, the majority of circulating FGF21 originates from the liver [9,10]. It has been demonstrated to enhance insulin sensitivity and lipoprotein profiles in obese rodents by enhancing catabolism in both white and brown adipose tissues (BAT) through interactions with a receptor complex of FGF receptor 1 (FGFR1) and the co-receptor β-klotho (KLB) [10,11]. Mechanistically, FGF21 promotes the browning of white adipose tissue (WAT) and induces the activation of BAT via the transcription factor CREB [12]. FGF21 also inhibits the SUMOylation of PPARγ, which enhances adipocyte differentiation and insulin-sensitizing activity [13]. Additionally, FGF21 boosts the uptake of glucose and the secretion of adiponectin in adipocytes [14,15]. These observations indicate that FGF21 significantly influences energy expenditure and metabolic processes in adipose tissue.

Elevated serum FGF21 levels have been observed in diet-induced obese (DIO) mice [16]. However, as obesity progresses, reduced FGF21 signaling may result from the downregulation of KLB, leading to “FGF21 resistance” [16]. In both humans and rodents, blood FGF21 levels rise significantly during late pregnancy [17,18], yet the biological function of this significance upregulation, particularly in GDM, remains unclear.

Pregnancy represents a physiological model of adaptive and reversible adipose tissue metabolism [6]. FGF21 is involved in regulating adipose tissue metabolism; however, its role in GDM remains largely unknown. In this study, we found that in GDM, aggravated adipose tissue insulin resistance and inflammation are associated with impaired FGF21 signaling. Mechanistically, pregnancy-related upregulation of FGF21 stimulates the differentiation of regulatory T cells (Tregs) and reduces adipose tissue inflammation via linolenic acid-mediated PGE2 synthesis; however, this process was disturbed in GDM. These findings underscore the critical role of FGF21 in metabolic reprogramming of adipose tissue during pregnancy and highlight FGF21 signaling as a promising target for GDM therapy.

## 2. Materials and Method

### 2.1. Animals

All animal procedures were carried out in accordance with the guidelines established by the Institutional Animal Care and Research Committee at Sichuan Agricultural University (No. 20230154).

Eight-week-old C57BL/6J mice were obtained from Vital River Laboratory Animal Technology Co. Ltd. (Beijing, China). The FGF21 KO mice were sourced from Jackson Laboratory (033846), and we generated *Klb^Adipoq^* KO mice by crossing β-Klotho gene-floxed mice (Cyagen, Guangzhou, China, CKOAI200115YK3) with adiponectin-Cre mice (Jackson Laboratory, Bar Harbor, ME, USA, 010803). We used *Klb^loxp/loxp^* littermates as controls in our experiments.

To induce gestational diabetes mellitus, we randomly divided 8-week-old female C57BL/6 mice into two groups. One group received a normal diet (ND) with 10% calories from fat (D12450J), and the other group was fed a high-fat and high-sucrose (HFHS) diet which consisted of 35% carbohydrate, 20% protein, and 45% fat (D12451); both diets were obtained from Research Diets, and the feeding continued for 8 weeks before mating. We cohoused confirmed fertile male mice with female mice at a 1:1 ratio, and the presence of a vaginal plug marked day 0.5 (E0.5) of pregnancy. All mice were maintained at room temperature (20–22 °C) on a 12 h light/dark cycle, with unrestricted access to food and water.

### 2.2. Glucose Tolerance Test

Following an overnight fast, a glucose tolerance test was performed by administering D-glucose intraperitoneally (1 g/kg body weight). Blood glucose levels were measured using glucose test strips at 0, 15, 30, 45, 60, 90, 105, and 120 min after injection by glucose test strips (Roche Diagnostics, Basel, Switzerland).

### 2.3. Assays and Kits

We measured serum FGF21 (MF2100; R&D Systems, Minneapolis, MN, USA) levels, insulin (KE10089; Proteintech, Rosemont, IL, USA) levels, and tissues’ PGE2 (ab287802, Abcam, Waltham, MA, USA) using commercial enzyme-linked immunosorbent assay (ELISA) kits following the manufacturer’s instructions.

### 2.4. Cell Culture

Primary CD4^+^ T cells were isolated from the spleen using a Naïve CD4^+^ T Cell Isolation kit (Cat# 130-104-453, Miltenyi Biotec, Bergisch Gladbach, Germany), and 4 × 10^5^ cells were seeded in 24-well plates. Plates were coated with 2 μg/mL anti-CD3 mAb (Clone# 145-2C11, Cat#553057, BD Biosciences, New York, NY, USA) and 2 μg/mL anti-CD28 (Clone# 37.51, Cat#553294, BD Biosciences) at 37 °C for 2 h and washed with PBS. Cells were also stimulated with 100 U/mL Recombinant Mouse IL-2 (Cat#402-ML, R&D systems) and 3 ng/mL Recombinant Mouse TGF-β (Cat#240-B, R&D systems), in the presence or absence of treatments (FGF21,100 μM or PGE2, 100 nM) for the indicated time. Cells were cultured at 37 °C with 5% CO_2_ for 3 d.

### 2.5. Flow Cytometry

Single-cell suspensions were incubated with Fc-receptor-blocking antibody (Cat#553141, BD Biosciences). Dead cells were excluded with Live/DEAD^®^ fixable viability stain 780 (Cat#65388BD, Biosciences, St. Durham, NC, USA). For intracellular transcription-factor staining, cells were fixed and permeabilized with BD Pharmigen^TM^ Transcription Factor Buffer Set (Cat#562574, BD Biosciences), cells were washed once with Stain buffer (Cat#554656, BD Biosciences), followed by staining with different antibodies. For analysis of Tregs, cells were triple-stained with antibodies: anti-mouse CD45-BV510 (Clone 30-F11, Cat#563891, BD Biosciences), anti-mouse CD3e-FITC (Clone 145-2C11, Cat#553061, BD Biosciences), anti-mouse CD4-BB700 (Clone RM4-5, Cat#566407, BD Biosciences), and anti-mouse CD25-BV421 (Clone 7D4, Cat#564571, BD Biosciences). Intracellular staining for transcription factor analysis was performed using BD Pharmigen^TM^ Transcription Factor Buffer Set (BD Biosciences, cat# 562574) as per manufacturer’s instructions before antibody staining with anti-mouse Foxp3-APC (Clone FJK-16s, Cat# 17-5773-82, eBioscience, San Diego, CA, USA). After staining, cells were analyzed with the BD Verse Cell Analyzer (BD Biosciences). Data were analyzed using FlowJo software version X.0.7 (Tree Star, Inc., Ashland, OR, USA).

### 2.6. Quantitative PCR (q-PCR) Analysis

Total RNA was extracted from uteri using TRIzol reagent (Thermo Fisher Scientific, Waltham, MA, USA) and purified with RNA mini columns (Takara Bio, Kusatsu, Japan). cDNA was synthesized from 1 μg of RNA, and qPCR was performed in a 10 μL reaction volume using SYBR Green. Primer sequences for the target genes are provided in the Supplementary Materials, Table S1.

### 2.7. Western Blot Analysis

Tissues were homogenized with a FastPrep-24^TM^ 5G (Santa Ana, CA, USA) in cold RIPA buffer containing protease inhibitors (Sigma-Aldrich, Burlington, MA, USA). The lysates were centrifuged for 30 min at 18,000× *g* and 4 °C, then subjected total protein lysates from WAT to SDS-PAGE. We electro-transferred the proteins onto a PVDF membrane and probed them with antibodies. Anti-phospho-ERK1/2 (9101) and anti-total ERK1/2 (9102) antibodies were obtained from Cell Signaling Technology (Danvers, MA, USA). The protein bands were visualized using enhanced chemiluminescence reagents and quantified with ImageJ software (Version 1.51j8).

### 2.8. Tissue Histology and Immunohistochemistry

For immunohistochemistry, tissues were harvested and fixed in 4% paraformaldehyde, then embedded in paraffin. We cut 5 µm sections and stained sections with CD68 (MCA1957, Bio-Rad Laboratories, Hercules, CA, USA) and used 3,3-diaminobenzidine for detection, examining the results under bright-field microscopy (Nikon 80i, Tokyo, Japan).

### 2.9. Statistical Analysis

We analyzed the data using GraphPad Prism 8 software (GraphPad Software, La Jolla, CA, USA). Comparisons between two groups were assessed using an unpaired Student’s *t*-test. Results are presented as mean values ± standard error of the mean (SEM). We determined statistical significance at a threshold of *p* < 0.05.

## 3. Results

### 3.1. Gestational Diabetes Mellitus Exhibits Aggravated Adipose Tissues Inflammation and FGF21 Signaling Defects

**Figure 1:**
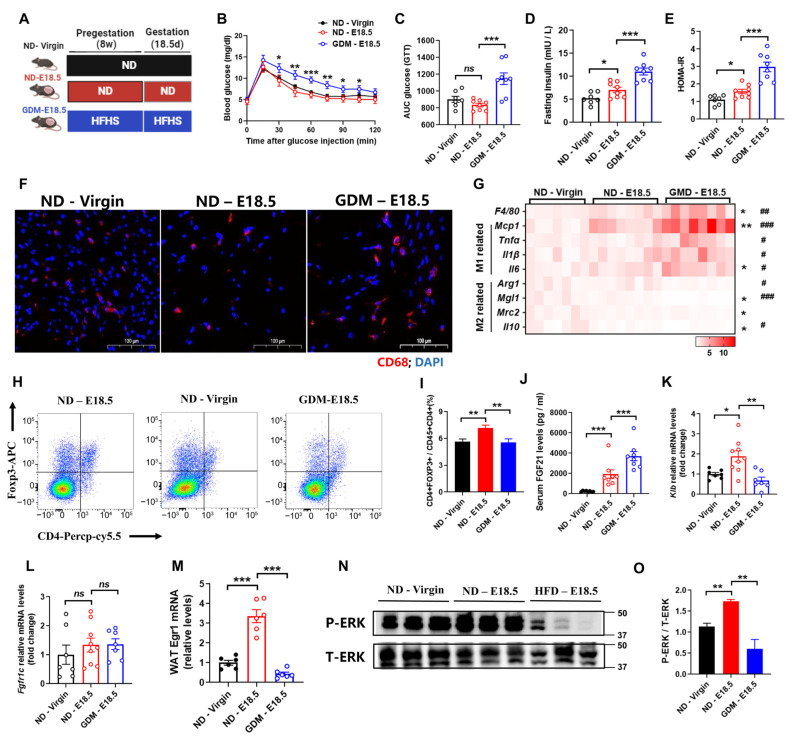
Gestational diabetes mellitus exhibits aggravated adipose tissue inflammation and FGF21 signaling defects. (**A**) A schematic illustration of the experimental design. C57BL/6 female mice were fed a normal diet (ND) or a high-fat and high-sucrose (HFHS) diet for 8 weeks before mating and pregnancy (E0.5 to E18.5, 18 days). (**B**,**C**) Changes to blood glucose and the area under the curve (AUC) in the glucose tolerance test. (**D**,**E**) Fasting serum insulin levels and the HOMA-IR. (**F**) Representative images of WAT stained with an anti-CD68 antibody (red). (**G**) RT-qPCR analysis for mRNA expression levels of *F4/80*, M1, and M2 macrophages-related genes. (**H**,**I**) Flow cytometry analysis for the numbers of Trges in WAT. Trges were defined as CD4^+^FOXP3^+^. (**J**) Serum FGF21 levels. (**K**,**L**) RT-qPCR analysis for mRNA expression levels of *Klb* and *Fgfr1c* in WAT (n = 7–10 per group). (**M**–**O**) RT-qPCR analysis for mRNA expression levels of *Egr1* (M) and Western blot analysis for Phosphorylated ERK1/2 (N, O) after tail vein injection of rmFGF21 (1 mg/kg). (n = 6 per group). Data are presented as means ± s.e.m. ND-Virgin vs. ND-E18.5, * *p* < 0.05, ** *p* < 0.01, *** *p* < 0.001. *ns* means no significant difference. ND—E18.5 vs. GDM—E18.5, ^#^ *p* < 0.05, ^##^ *p* < 0.01, ^###^ *p* < 0.001.

To evaluate the role of FGF21 in gestational diabetes mellitus (GDM), we collected visceral fat samples from three groups: non-pregnant chow diet female mice (ND-Virgin), pregnant chow diet female mice (ND-E18.5), and pregnant high-fat high-sucrose diet female mice (GDM-E18.5) (Figure 1A). Consistent with prior studies [19], GDM-E18.5 mice displayed impaired glucose tolerance and reduced insulin sensitivity compared to ND-E18.5 mice (Figure 1B–E). Notably, we observed increased macrophage accumulation during pregnancy, which was significantly heightened in GDM mice (Figure 1F). Real-time PCR analysis confirmed increased expression of M1 macrophage markers (*Mcp1*, *Tnfα*, *Il1β*, *Il6*) and decreased M2 markers (*Arg1*, *Mgl1*, *Il10*) in GDM mice compared to ND-E18.5 (Figure 1G). Furthermore, we found that the percentage of regulatory T cells (Tregs) increased with gestation; this increase was diminished in GDM mice (Figure 1H,I), suggesting that GDM promotes adipose tissue inflammation.

Elevated serum FGF21 levels were noted in DIO and pregnant mice [16,18]. To assess the function of the biological significance of these complex and variable changes in FGF21 levels, we measured the serum FGF21 levels in GDM mice. The results showed that pregnant mice exhibited higher circulating FGF21, which was further elevated in GDM mice (Figure 1J). To characterize FGF21’s biological role during pregnancy, we analyzed expression levels of FGF21 receptor 1c (*Fgfr1c*) and *Klb*. While *Fgfr1c* levels were similar in visceral white adipose tissue (WAT) (Figure 1K), *Klb* mRNA was significantly higher in ND-E18.5 mice compared to ND-Virgin mice (Figure 1L), indicating strengthened FGF21 signaling during healthy pregnancy. However, *Klb* expression decreased in the visceral fat of GDM mice, suggesting a state of “FGF21 resistance”. To further confirm this, the mice were treated with a single bolus of recombinant FGF21 (rmFGF21). Following the treatment, we observed significant increases in *Egr1* mRNA and phosphorylation of ERK_1/2_ in the visceral adipose tissue of pregnant mice, further confirming enhanced FGF21 signaling (Figure 1M,N). In contrast, GDM mice showed a weakened response, indicating that pregnancy increases FGF21 sensitivity (Figure 1M,N), while GDM induces “FGF21 resistance”, likely contributing to aggravated adipose tissue inflammation in GDM mice.

### 3.2. FGF21 Is Required for Alleviates Adipose Tissues Inflammation During Pregnancy

**Figure 2:**
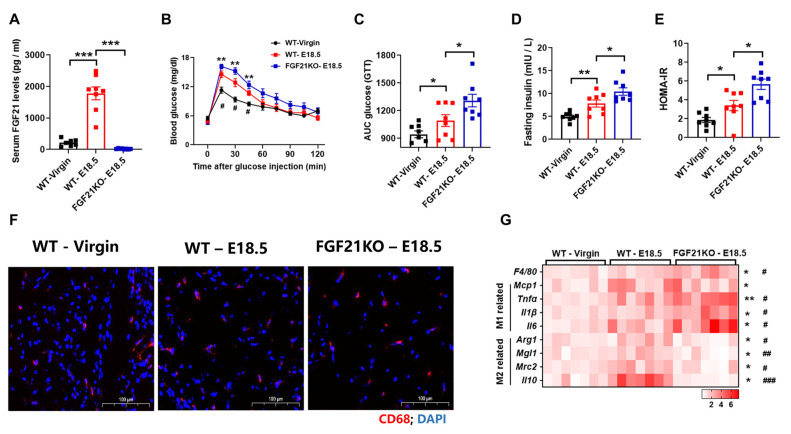
FGF21 is required for alleviating adipose tissue inflammation during pregnancy. (**A**) Serum FGF21 levels. (**B**,**C**) Changes to blood glucose and the area under the curve (AUC) in the glucose tolerance test. (**D**,**E**) Fasting serum insulin levels and the HOMA-IR. (**F**) Representative images of WAT stained with an anti-CD68 antibody (red). (**G**) RT-qPCR analysis for mRNA expression levels of *F4/80*, M1, and M2 macrophages-related genes. (n = 7–8 per group). Data are presented as means ± s.e.m. WT-Virgin vs. WT-E18.5, * *p* < 0.05, ** *p* < 0.01, *** *p* < 0.001. WT—E18.5 vs. FGF21KO—E18.5, ^#^ *p* < 0.05, ^##^ *p* < 0.01, ^###^ *p* < 0.001.

To further investigate FGF21’s involvement in pregnancy-induced adipose tissue (AT) phenotypes, we examined AT inflammation in wild-type (WT) and FGF21-knockout (FGF21KO) mice during late pregnancy. Serum FGF21 levels were increased in WT mice during pregnancy, but not in FGF21KO mice (Figure 2A). Glucose tolerance tests showed impairment in WT mice, and was further exacerbated in FGF21-KO mice (Figure 2B–E). While pregnancy increased AT inflammation in both groups, the inflammation was more severe in FGF21-KO mice (Figure 2F), aligning with M1 and M2 macrophage marker gene expression analyzed via real-time PCR (Figure 2G). These results suggest that FGF21 deficiency exacerbates AT inflammation during pregnancy.

### 3.3. FGF21 Promotes Tregs Differentiation Through Linolenic Acid-Mediated PGE2 Synthesis in Adipocytes

**Figure 3:**
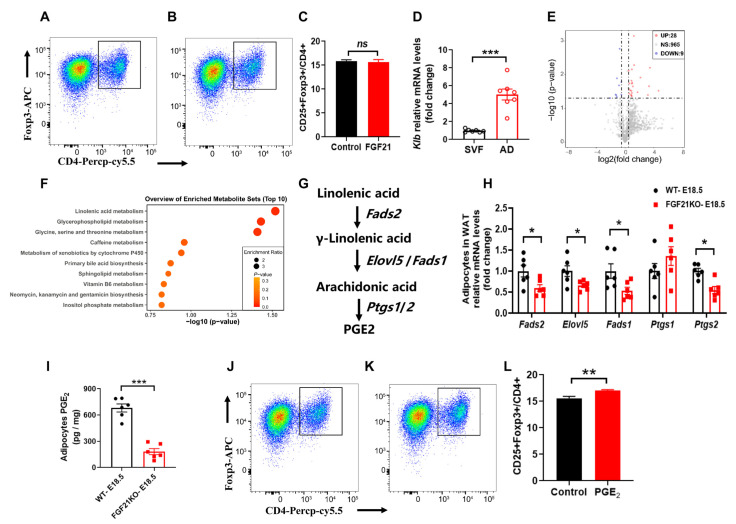
FGF21 promotes Tregs differentiation through linolenic acid-mediated PGE2 synthesis in adipocytes. (**A**–**C**) Primary CD4^+^ T cells were purified from spleen for these studies. The number of Tregs after FGF21 treatment (100 μM). (**D**) RT-qPCR analysis for mRNA expression levels of *Klb* in stromal vascular fraction (SVF) and mature adipocyte (AD) fraction isolated from WAT at E18.5 of pregnancy. (**E**) A volcano plot showing significantly changed metabolites in the mature adipocyte between WT and FGF21KO mice at E18.5 of pregnancy. (**F**) Metabolic pathways showing significant changes in linolenic acid metabolism. (**G**,**H**) The linolenic acid metabolic pathway to synthesis of PGE2 and the rate-limiting enzyme mRNA expression levels in mature adipocyte. (**I**) The PGE2 levels in WAT at E18.5 of pregnancy as determined by ELISA. (**J**–**L**) The number of Tregs after PGE2 treatment (100 nM). Data are presented as means ± s.e.m. * *p* < 0.05, ** *p* < 0.01, *** *p* < 0.001. *ns* means no significant difference.

Tregs are critical anti-inflammatory immune cells in AT [20,21], and the increased inflammation in FGF21-KO mice implies that FGF21 may regulate Tregs activation. We extracted primary CD4^+^ T cells from the spleen to assess the direct effects of FGF21 on Tregs’ differentiation and proliferation. However, rmFGF21 treatment did not significantly affect Tregs’ differentiation or proliferation (Figure 3A,B), indicating that Tregs may not be sensitive to FGF21’s direct effects. In alignment with this, *Klb* expression was primarily found in mature adipocytes, not in the stromal vascular fraction containing most immune cells in AT (Figure 3C). There is growing evidence that changes in metabolite abundance can activate immune cells in AT; we isolated mature adipocytes from WT and FGF21-KO mice visceral fat on day 18.5 of pregnancy, and conducted untargeted metabolomics analyses.

The untargeted metabolomics analyses revealed distinct metabolite profiles between the two groups, with FGF21-KO leading to 28 upregulated and 9 downregulated differentially enriched metabolites (Figure 3D). Pathway analysis indicated that linolenic acid metabolism, crucial for regulating inflammation and energy expenditure by controlling synthesis of prostaglandins (PGs), was significantly affected (Figure 3E). The linolenic acid metabolic pathway to synthesis of PGE2 contains five rate-limiting enzymes including fatty acid desaturase 1/2 (encoded by *Fads1/2*), fatty acid elongase 5 (encoded by *Elovl5*), and prostaglandin-endoperoxide synthase 1/2 (encoded by *Ptgs1/2*) (Figure 3F). Key enzymes in this pathway showed decreased expression in FGF21-KO adipocytes (Figure 3G), correlating with reduced PGE2 levels confirmed by ELISA (Figure 3H). These results suggest that FGF21 mediates its anti-inflammatory effects through PGE2 production in adipocytes. To validate the ability of PGE2 on Tregs activation, we tested its effects on primary CD4^+^ T cells, and found that PGE2 significantly increased the number of CD4^+^Fxop3^+^ Tregs, without affecting the proliferation of total Tregs (Figure 3I,J), suggesting that PGE2 promotes differentiation into Tregs.

### 3.4. Deficiency of FGF21 Signaling in Adipocytes Increases Adipose Tissues Inflammation During Pregnancy

**Figure 4:**
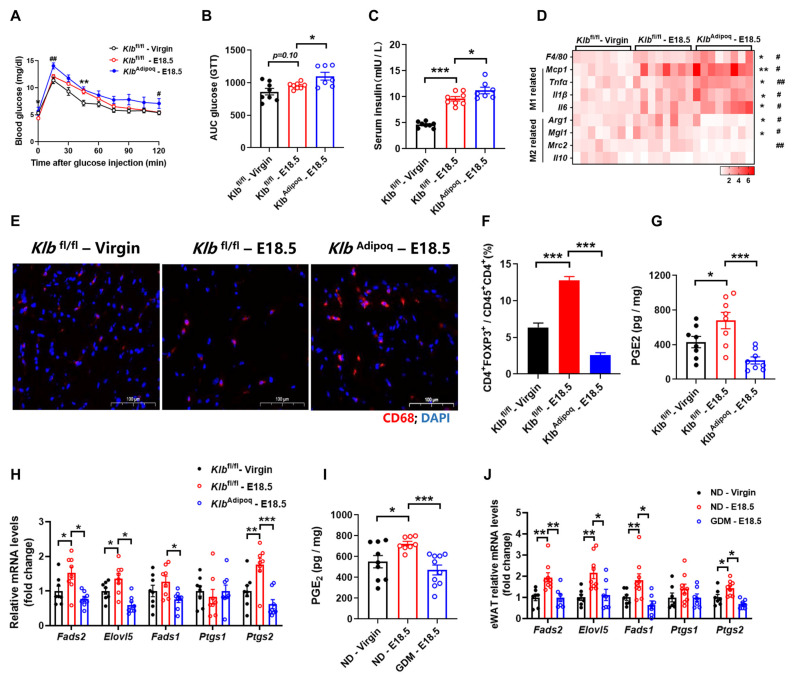
Deficiency of FGF21 signaling in adipocytes increases adipose tissues inflammation during pregnancy. (**A**,**B**) Glucose tolerance test, and area under curve. (**C**) Serum insulin levels measured by ELISA. (**D**) RT-qPCR analysis for mRNA expression levels of *F4/80*, M1 and M2 macrophages related genes. (**E**) Representative images of WAT stained with anti-CD68 antibody (red). (**F**) Flow cytometry analysis for numbers of Trges in WAT. Trges were defined as CD4^+^FOXP3^+^. PGE2 levels and synthesis rate-limiting enzyme mRNA expression levels in WAT at E18.5 of pregnancy from *Klb*^Adipoq^ (**G**,**H**) and GDM mice (**I**,**J**). Data are presented as means ± s.e.m. *Klb*^fl/fl^-Virgin vs. *Klb*^fl/fl^—E18.5, * *p* < 0.05, ** *p* < 0.01, *** *p* < 0.001. *Klb*^fl/fl^—E18.5 vs. *Klb*^Adipoq^—E18.5, ^#^ *p* < 0.05, ^##^ *p* < 0.01.

To further characterize the physiological role of adipocyte FGF21 signaling in regulating PGE2 synthesis and AT inflammation, we generated adipocyte-specific KLB-KO mice by crossing *Klb*–floxed mice and adiponectin-cre mice. As expected, glucose tolerance was impaired in *Klb*^Adipoq^ mice compared with littermate *Klb*^fl/fl^ (wild type) mice (Figure 4A–C). To further investigate the impact of the deletion of adipose FGF21 signaling on immune cell activation in visceral WAT, we monitored the AT inflammation at E18.5. The number of CD68^+^ cells was increased in pregnant *Klb*^fl/fl^ mice, compared with no-pregnancy mice; furthermore, the number of CD68^+^ cells in *Klb*^Adipoq^ mice increased more significantly than in the control pregnancy littermates (Figure 4D). Consistent with this, we found that F4/80 and the marker of M1 macrophages including *Mcp1*, *Tnfα*, *Il1β*, and *Il6* were increased; however, the marker of M2 macrophages including *Arg1*, *Mgl1*, and *Mrc2* were decreased in *Klb*^Adipoq^ mice, which were compared with pregnant *Klb*^fl/fl^ mice (Figure 4E). Further cell fractional analysis showed a reduced proportion of Tregs in CD4^+^ cells in the visceral WAT of *Klb*^Adipoq^ mice (Figure 4F). These findings collectively suggest that a deficiency in FGF21 signaling in adipocytes exacerbates adipose tissue inflammation during pregnancy.

Moreover, we also found that AT PGE2 levels were higher in pregnant *Klb*^fl/fl^ mice compared to non-pregnant controls, while *Klb*^Adipoq^ mice showed significantly lower PGE2 levels in AT than control mice on E18.5 (Figure 4G,H). Real-time PCR analysis confirmed decreased expression of PGE2 synthesis markers in GDM mice, similar to FGF21-KO and *Klb*^Adipoq^ mice (Figure 4I,J). Combined with GDM mice showing an “FGF21 resistance” phenotype, these results indicate that defects in FGF21 signaling within adipose tissue exacerbate inflammation and insulin resistance in GDM.

## 4. Discussion

Despite extensive research on the metabolic functions of FGF21, its role in immune regulation remains controversial. Our study provides evidence that FGF21 signaling in adipocytes promotes the differentiation of regulatory T cells (Tregs), which is crucial for alleviating pregnancy-induced inflammation in adipose tissues. The anti-inflammatory effect of FGF21 may occur through linolenic acid-mediated PGE2 synthesis. These findings reinforce the concept of FGF21 as a physiological integrator of metabolism and immunity, facilitating communication between mature adipocytes and immune cells in white adipose tissue (WAT).

During pregnancy, maternal metabolic reprogramming occurs alongside moderate inflammation in adipose tissues [5,22]. FGF21, a key metabolic regulator involved in glucose homeostasis and insulin sensitivity, increases during late pregnancy [18,23]. Thus, we hypothesized that elevated FGF21 signaling in healthy pregnancies alleviates adipose tissue inflammation. Indeed, inflammation was exacerbated in both FGF21-KO and adipocyte-specific KLB-KO mice during pregnancy. Tregs, essential anti-inflammatory cells in adipose tissue, increase in circulation, the spleen, and the placenta as pregnancy progresses [20,24]. While some studies suggest that FGF21 may enhance cholesterol biosynthesis in CD8^+^ T cells via FGFR1-KLB receptors [25], we found that FGF21 signaling does not directly affect macrophages and Tregs. Consistent with previous research [26], Klb expression levels in immune cells were very low, indicating that FGF21’s role in immune regulation operates through indirect pathways.

In mouse adipocytes, FGF21 promotes glucose uptake, aids in lipid disposal, and triggers browning and adaptive thermogenesis [14,23,27]. With KLB highly expressed in adipocytes [28,29], we hypothesized that FGF21’s anti-inflammatory effects are mediated by adipocyte metabolism. Indeed, we found that FGF21 directly acts on adipocytes to promote PGE2 synthesis via linolenic acid. Prostaglandins are key regulators of adipose inflammation and insulin resistance [30,31,32,33]. Our study demonstrated that PGE2 enhances Tregs’ differentiation, supported by recent findings that the immune-suppressive effects of adipose-resident Tregs are prompted by the metabolite 15-keto-PGE2. Interestingly, FGF21 increased key enzymes for PGE2 synthesis from linolenic acid, including *Fads1/2* and *Elovl5*, while having no effect on *Ptgs2*. These results suggest that the anti-inflammatory effects in adipose tissues are coordinated by multiple factors, with FGF21 mediating crosstalk between mature adipocytes and immune cells in WAT.

## 5. Conclusions

GDM is significantly associated with various pregnancy complications, including prematurity, hypoglycemia, macrosomia, and increased metabolic risk for offspring in adulthood [2,3]. In our study, we observed that FGF21 signaling in visceral fat was strengthened during healthy pregnancy. However, in GDM, *Klb* expression decreased, indicating a state of “FGF21 resistance”, which reduced the PGE2-induced accumulation of Tregs in adipose tissues. Given that GDM is linked to more severe adipose inflammation, our findings raise the possibility that FGF21 signaling and its downstream metabolites may serve as alternative therapeutic strategies for GDM, particularly in cases of FGF21 resistance. It is worth noting that further large-scale, multicenter human studies are needed to clarify the significance of FGF21 signaling and FGF21 resistance in human GDM.

## Data Availability

All data and datasets supporting the conclusions of this study are either included in the paper, or are available upon reasonable request from the corresponding author.

## Supplementary Materials

The following supporting information can be downloaded at: https://www.mdpi.com/article/10.3390/nu16223826/s1, Table S1. Sequence of RT-PCR primers.
