# Supplementary material for: Adipocyte FGF21 Signaling Defect Aggravated Adipose Tissue Inflammation in Gestational Diabetes Mellitus
Source: Nutrients. 2024 Nov 7;16(22):3826. doi: 10.3390/nu16223826 (PMC11597770; doi:10.3390/nu16223826)
Supplement: Supplementary file 1 [file nutrients-16-03826-s001.zip › nutrients-3273753-supplementary.pdf]

Supplementary Table 1. Sequence of the RT-PCR primers.

Name	Forward	Reverse
β-actin	GGCTGTATTCCCCTCCATCG	CCAGTTGGTAACAATGCCATGT
F4/80	CTTTGGCTATGGGCTTCCAGTC	GCAAGGAGGACAGAGTTTATCGTG
Mcp1	CCACTCACCTGCTGCTACTCA	TGGTGATCCTCTTGTAGCTCTCC
Il6	GATGGATGCTACCAAACTGGA	TCTGAAGGACTCTGGCTTTG
Arg1	GGAATCTGCATGGGCAACCTGTGT	AGGGTCTACGTCTCGCAAGCCA
Mgl1	TGAGAAAGGCTTTAAGAACTGGG	GACCACCTGTAGTGATGTGGG
Mrc2	TACAGCTCCACGCTATGGATT	CACTCTCCCAGTTGAGGTACT
Tnfα	CAGCCTCTTCTCATTCCTGC	GGTCTGGGCCATAGAACTGA
Il1β	CTGGTGTGTGACGTTCCCATTA	CCGACAGCACGAGGCTTT
Il10	ATCGATTTCTCCCCTGTGAA	TGTCAAATTCATTCATGGCCT
Fads2	AAGGGAGGTAACCAGGGAGAG	CCGCTGGGACCATTTGGTAA
Elovls5	ATGGAACATTTCGATGCGTCA	GTCCCAGCCATACAATGAGTAAG
Fads1	AGCACATGCCATACAACCATC	TTTCCGCTGAACCACAAAATAGA
Ptgs1	GTGCTGGGGCAGTGCTGGAG	TGGGGCCTGAGTAGCCCGTG
Ptgs2	CTCACGAAGGAACTCAGCACT	TAGAATCCAGTCCGGGTACAGT
Klb	CAACCCACTCCCATCTCGG	AGCACAGCTCAGCGTAGTCC
Fgfr1c	ACTCTGCGCTGGTTGAAAAAT	GGTGGCATAGCGAACCTTGTA
Egr1	TCGGCTCCTTTCCTCACTCA	CTCATAGGGTTGTTCGCTCGG
